# Modulation of Mitochondrial Outer Membrane Permeabilization and Apoptosis by Ceramide Metabolism

**DOI:** 10.1371/journal.pone.0048571

**Published:** 2012-11-30

**Authors:** António Rego, Margarida Costa, Susana Rodrigues Chaves, Nabil Matmati, Helena Pereira, Maria João Sousa, Pedro Moradas-Ferreira, Yusuf A. Hannun, Vítor Costa, Manuela Côrte-Real

**Affiliations:** 1 Departamento de Biologia, Centro de Biologia Molecular e Ambiental, Universidade do Minho, Braga, Portugal; 2 Instituto de Biologia Molecular e Celular, Universidade do Porto, Porto, Portugal; 3 Departamento de Biologia Molecular, Instituto de Ciências Biomédicas Abel Salazar, Universidade do Porto, Porto, Portugal; 4 Stony Brook Cancer Center, Stony Brook University, Health Science Center, Stony Brook, New York, United States of America; National Institutes of Health, United States of America

## Abstract

The yeast *Saccharomyces cerevisiae* undergoes a mitochondrial-dependent programmed cell death in response to different stimuli, such as acetic acid, with features similar to those of mammalian apoptosis. However, the upstream signaling events in this process, including those leading to mitochondrial membrane permeabilization, are still poorly characterized. Changes in sphingolipid metabolism have been linked to modulation of apoptosis in both yeast and mammalian cells, and ceramides have been detected in mitochondria upon apoptotic stimuli. In this study, we aimed to characterize the contribution of enzymes involved in ceramide metabolism to apoptotic cell death induced by acetic acid. We show that *isc1*Δ and *lag1*Δ mutants, lacking inositol phosphosphingolipid phospholipase C and ceramide synthase, respectively, exhibited a higher resistance to acetic acid that was associated with lower levels of some phytoceramide species. Consistently, these mutant cells displayed lower levels of ROS production and reduced mitochondrial alterations, such as mitochondrial fragmentation and degradation, and decreased translocation of cytochrome *c* into the cytosol in response to acetic acid. These results suggest that ceramide production contributes to cell death induced by acetic acid, especially through hydrolysis of complex sphingolipids catalyzed by Isc1p and *de novo* synthesis catalyzed by Lag1p, and provide the first *in vivo* indication of its involvement in mitochondrial outer membrane permeabilization in yeast.

## Introduction

Apoptosis is an evolutionary conserved type of programmed cell death (PCD) that is crucial for normal tissue homeostasis and development. For many years, apoptosis was assumed to be restricted to multicellular organisms. However, it is now established that several stimuli can induce in unicellular organisms the same typical apoptotic markers that are observed in multicellular organisms. An apoptotic-like cell death can greatly benefit a population of unicellular organisms, for instance by eliminating virus-infected and damaged cells, and releasing nutrient sources for the fittest individuals [1]. The research community has however also taken advantage of this simplified model system to elucidate several aspects of apoptosis regulation in mammalian cells, namely of the intrinsic mitochondrial pathway.

We have previously shown that acetic acid triggers a mitochondria-mediated apoptotic pathway in *Saccharomyces cerevisiae* associated with chromatin condensation, exposure of phosphatidylserine to the outer leaflet of the plasma membrane, formation of DNA strand breaks, accumulation of mitochondrial reactive oxygen species (ROS) and mitochondrial outer membrane permeabilization (MOMP) and subsequent release of cytochrome *c* [2], [3] and yeast apoptosis-inducing factor [4]. Acetic acid-induced apoptosis has also been linked with mitochondrial fragmentation and degradation [5], [6]. We also found that the ADP/ATP carrier is involved in MOMP and cytochrome *c* release [7], and that Pep4p, an orthologue of the mammalian cathepsin D (CatD), is released from the vacuole into the cytosol during acetic acid-induced apoptosis to act in mitochondrial degradation [6].

However, there is little evidence regarding upstream signalling events leading to apoptosis in response to acetic acid. Here, we hypothesized that ceramide, an important modulator of apoptosis in mammalian cells, may play a role in this process. Indeed, sphingolipids have emerged as important bioactive molecules in cell regulation, in addition to being critical structural components of cellular membranes. Ceramide, the central core lipid in the metabolism of sphingolipids, is produced by acylation of a long chain sphingoid base (sphingosine) in a *de novo* biosynthetic pathway or via hydrolysis of complex sphingolipids, such as sphingomyelin, by mammalian sphingomyelinases. The accumulation of ceramides by activation of sphingomyelinases has been observed in response to a variety of stimuli, including heat stress and oxidants [8], [9]. Whereas sphingosine and ceramide induce cell death, other sphingolipids such as sphingosine-1-phosphate promote cell survival or proliferation [10]. The effects of ceramide are mediated by activation of protein kinases or phosphatases, as well as through interaction with components of cell signalling pathways [11], [12]. In mammalian cells, ceramide also increases ROS production by directly inhibiting the mitochondrial complex III of isolated mitochondria [13] and increasing the permeability of mitochondrial membranes to cytochrome *c* [14]. More recently, it has also been found that the two sphingolipid products sphingosine-1-phosphate and hexadecenal act as specific cofactors of BAX/BAK activation, lowering the threshold for MOMP and apoptosis-associated cytochrome *c* release [15].

**Figure 1:**
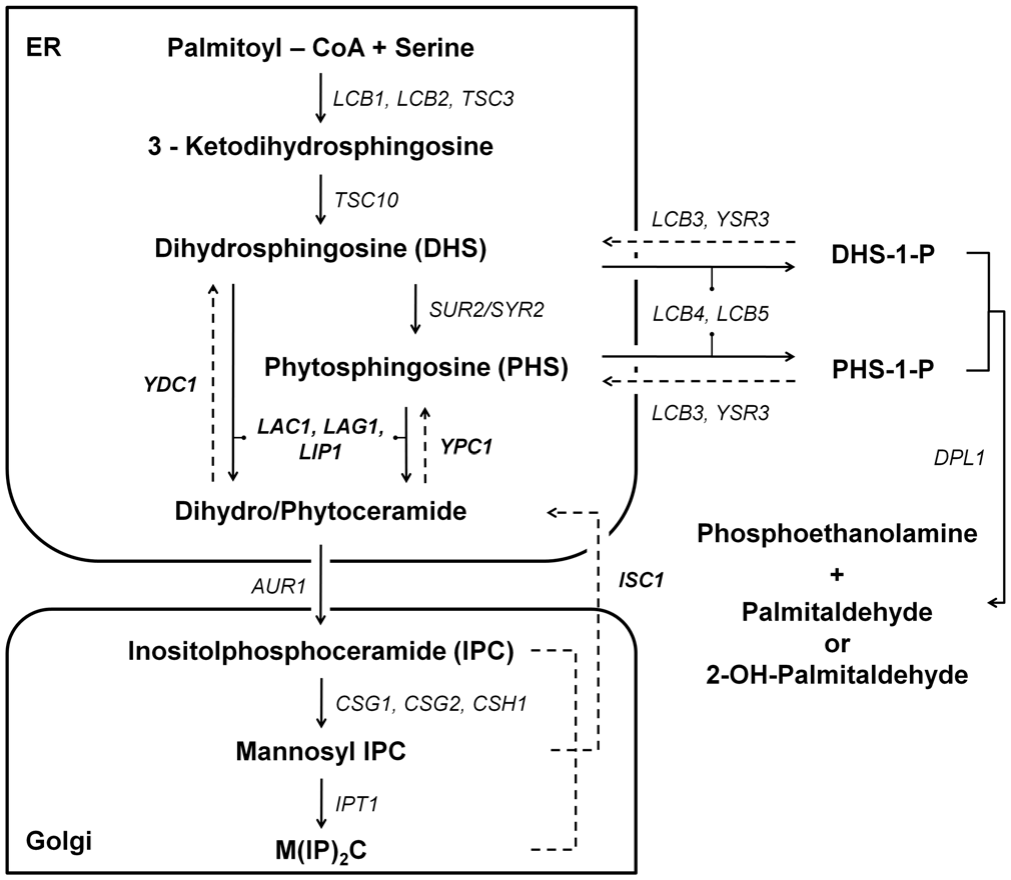
Sphingolipids metabolism in yeast. Schematic overview of yeast sphingolipid metabolism displaying the metabolic intermediates, genes involved and cellular locations of the enzymatic reactions.

In yeast, like in mammalian cells, ceramide levels increase in response to diverse stress treatments [16], [17]. Sphingolipid metabolism in *S. cerevisiae* is similar to that of its mammalian counterpart, but yeast use ceramide to synthesise inositolphosphosphingolipids instead of sphingomyelin. In yeast, dihydroceramide and phytoceramide can be produced by *de novo* biosynthesis through acylation of a long chain sphingoid base catalysed by the Lag1p or Lac1p catalytic subunits of ceramide synthase, or by degradation of inositolphosphosphingolipids mediated by Isc1p. Moreover, dihydroceramide and phytoceramide can be hydrolysed by Ydc1p or Ypc1p ceramidases [18] (Figure 1).

The *ISC1* gene codes for inositol phosphosphingolipid phospholipase C, orthologue of mammalian neutral sphingomyelinases-2 [19]. Isc1p produces ceramides from complex sphingolipids and is post-translationally regulated by translocation into mitochondria when aerobic respiration is induced (e.g., in the post-diauxic phase when cells are grown in glucose) [20]. It is required for normal mitochondrial function [21], [22] and modulates cellular responses to osmostress [23], heat stress [24], genotoxic agents [25], oxidative stress, and aging [26]. Isc1p is an upstream regulator of Sit4p, the catalytic subunit of ceramide-activated protein phosphatase type 2A, and of Hog1p, the mitogen-activated protein kinase (MAPK) of the high osmolarity glycerol (HOG) pathway, and deletion of *SIT4* or *HOG1* in *isc1*Δ cells abolishes the premature ageing and oxidative stress sensitivity of this strain by suppressing mitochondrial dysfunction [27], [28]. The *LAG1* gene, orthologue of mammalian longevity assurance gene (LASS1), encodes ceramide synthase and is known to regulate replicative life-span in *S. cerevisiae* [29], [30].

Until now, few studies were performed in yeast to address the involvement of sphingolipids in apoptosis. Siskind and coworkers observed that recombinant Bcl-xL or CED-9, homologues of Bcl-2 proteins, disassembled ceramide channels in isolated mitochondria of yeast cells [31]. In another study, overexpression of Ydc1p ceramidase triggered vacuolar and mitochondrial fragmentation and dysfunction, shortened chronological lifespan, and increased apoptosis [32]. Moreover, *ISC1* deletion is associated with up-regulation of the iron regulon and high levels of ROS, and increases apoptotic cell death triggered by hydrogen peroxide or associated with cell aging [26]. It was also recently reported that exogenous N-acetyl-D-sphingosine (C_2_-Ceramide) triggers a mitochondria-mediated cell death process [33].

In this study, we assessed the role of enzymes involved in ceramide metabolism in acetic acid-induced PCD. We found that absence of two enzymes involved in ceramide production (Isc1p and Lag1p) led to increased resistance to acetic acid-induced PCD that was associated with lower levels of some phytoceramide species and reduced mitochondrial dysfunction, namely ROS production, mitochondrial fragmentation and degradation, and cytochrome *c* release into the cytosol. Our data suggest that acetic acid may increase ceramide levels through hydrolysis of complex lipids and *de novo* synthesis catalyzed by Isc1p and Lag1p, respectively, leading to mitochondrial dysfunction and consequently apoptosis. These results show the conservation of the involvement of ceramide metabolism in apoptosis, and provide the first *in vivo* indication of its involvement in MOMP in yeast.

## Results

### Deletion of *ISC1* or *LAG1* increased cellular resistance to acetic acid

**Figure 2:**
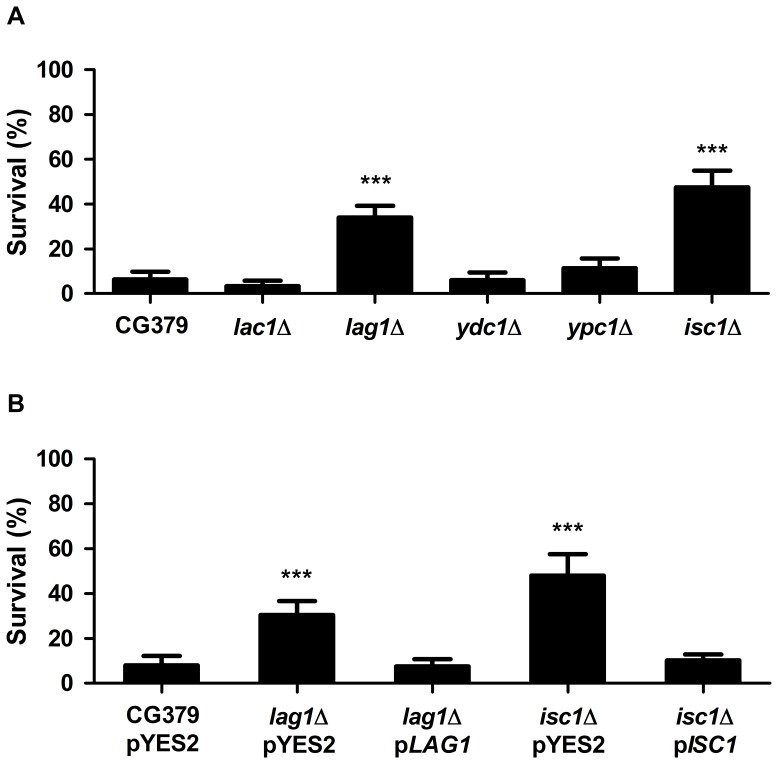
Acetic acid resistance in *S. cerevisiae*. (A) CG379 (wild-type), *lac1*Δ, *lag1*Δ, *ydc1*Δ, *ypc1*Δ and *isc1*Δ strains, or (B) CG379 pYES2 (empty vector), *lag1*Δ transformed with pYES2 or p*LAG1*, and *isc1*Δ transformed with pYES2 or p*ISC*1. Cells were exposed to 180 mM acetic acid, pH 3.0, for 200 min. Cell viability was determined by standard dilution plate counts and expressed as a percentage of c.f.u on YPD plates. Values are mean ± SD of at least three independent experiments. Values significantly different from CG379 strain: *** P<0.001, One-way ANOVA and Tukey Test.

In order to characterize the relative contribution of *de novo* biosynthesis *versus* catabolism of sphingolipids to acetic acid-induced apoptotic cell death, *S. cerevisiae* CG379 strains unable to generate ceramides by degradation of inositolphosphosphingolipids (*isc1*Δ), or by *de novo* biosynthesis (*lag1*Δ and *lac1*Δ), or unable to breakdown ceramides (*ydc1*Δ and *ypc1*Δ), were constructed by homologous recombination. Yeast cells were grown to exponential phase in SC Gal medium and exposed to 180 mM acetic acid, pH 3.0, for 200 min. As seen in Figure 2A, deletion of *ISC1* or *LAG1* increased the resistance of yeast cells to acetic acid, whereas deletion of *LAC1*, *YDC1* or *YPC1* had no effect. Acetic acid-induced cell death in the different strains was not associated with significant loss of plasma membrane integrity as measured by propidium iodide (PI) staining, indicating this in an active process (data not shown). Expression of *LAG1* and *ISC1* from a multi-copy plasmid (pYES2-*LAG1* and pYES2-*ISC1*) in *lag1*Δ and *isc1*Δ cells, respectively, reverted the high resistance of these strains to acetic acid, confirming the observed phenotypes were due to disruption of these genes (Figure 2B).

### The resistance of *isc1*Δ and *lag1*Δ cells to acetic acid is related with lower levels of intracellular ROS

**Figure 3:**
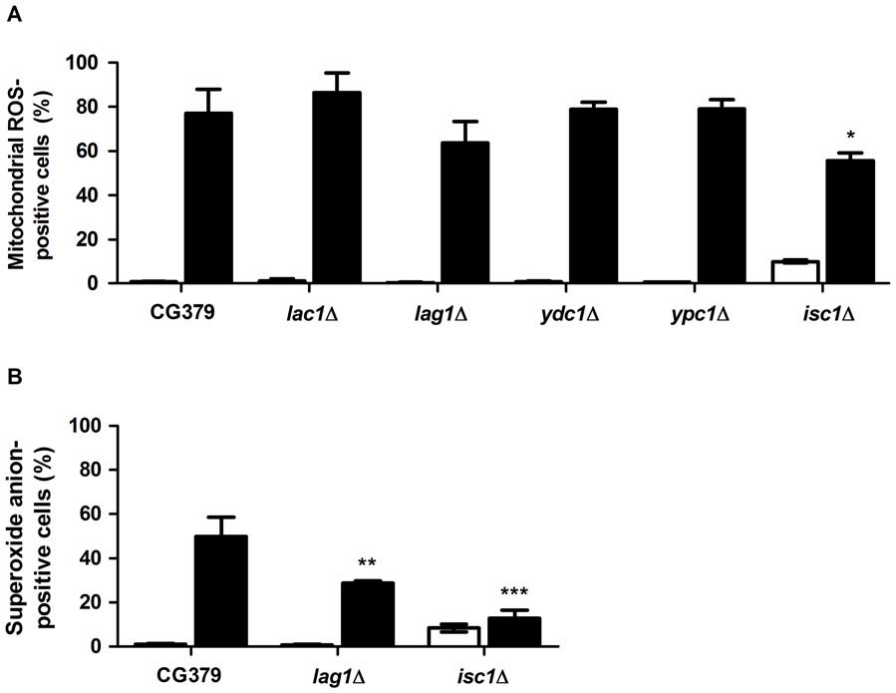
Intracellular ROS. Levels of mitochondrial ROS (A) and superoxide anion (B) in the indicated *S. cerevisiae* strains exposed to 180 mM acetic acid, pH 3.0, for 0 min (white columns) and 200 min (black columns), using MitoTracker Red CM-H_2_XRos (A) and DHE (B), respectively. Values are mean+SD of at least three independent experiments. Values significantly different from CG379: * P<0.05, ** P<0.01 and *** P<0.001, One-way ANOVA and Tukey Test.

Apoptosis is in many cases associated with the production of ROS in a wide variety of organisms, including yeast [34], [35]. ROS comprises the superoxide anion, which is mainly generated in mitochondria, hydroxyl radicals, and hydrogen peroxide. When the levels of ROS exceed the antioxidant capacity of the cells, homeostasis is disrupted and molecules such as lipids, proteins, and nucleic acids are oxidized, compromising survival [36]. We therefore determined the involvement of sphingolipid metabolism in the production of ROS in response to exposure to acetic acid. Untreated cells or cells treated with acetic acid were labeled with MitoTracker Red CM-H_2_XRos or Dihydroethidium (DHE), to detect mitochondrial free radicals and superoxide anions, respectively, and analyzed by flow cytometry (Figure 3). In wild type cells, exposure to acetic acid increased the percentage of ROS-positive cells assessed using any of the probes. This increase was significantly lower in *isc1*Δ cells (using either MitoTracker Red CM-H_2_XRos or DHE) and in *lag1*Δ cells (using the DHE probe), in agreement with their resistance phenotype. These results suggest that sphingolipids generated by Isc1p and Lag1p play a role in acetic acid-induced ROS production.

In order to explore whether the phenotypes described above were associated with oxidative damage, protein carbonylation was analyzed by immunodetection in both wild-type and mutant cells (Figure S1A). Hydrogen peroxide was used as positive control, since there is a correlation between H_2_O_2_ exposure and accumulation of carbonylated proteins [37], [38]. In accordance with our previous results [6], overall levels of protein carbonylation in untreated and acetic acid-treated cells were similar, and much lower than those observed in cells treated with H_2_O_2_. However, cells treated with H_2_O_2_ had the same total protein profile as untreated cells, whereas in cells treated with acetic acid there was an enrichment in three protein bands, while others were greatly decreased or disappeared altogether (Figure S1B). One explanation may come from a previous report indicating transient proteasome activation is required for acetic acid-induced cell death [39]. It is possible there is an increase in carbonylation of these three proteins, or that the increased signal is merely a reflection of their increased levels (observed by silver staining). In any case, deletion of *ISC1* or *LAG1* had no effect on the carbonylation profile observed, indicating the increased resistance of these mutants to acetic acid is not associated with changes in protein oxidation.

### Deletion of *ISC1* or *LAG1* alters mitochondrial morphology and delays mitochondrial degradation in response to acetic acid

Mitochondria are essential organelles that exist in dynamic networks, and often change their localization and shape during stress conditions [40]. Acetic acid triggers a mitochondria-dependent apoptotic pathway in yeast, associated with typical mitochondrial markers such as hyperpolarization followed by depolarization of the mitochondrial membrane and release of cytochrome *c* into the cytosol, as well as mitochondrial fragmentation and degradation [2], [6]. Since the *isc1*Δ and *lag1*Δ mutants were more resistant to acetic acid than wild-type cells, we assessed whether they still exhibited these apoptotic markers.

**Figure 4:**
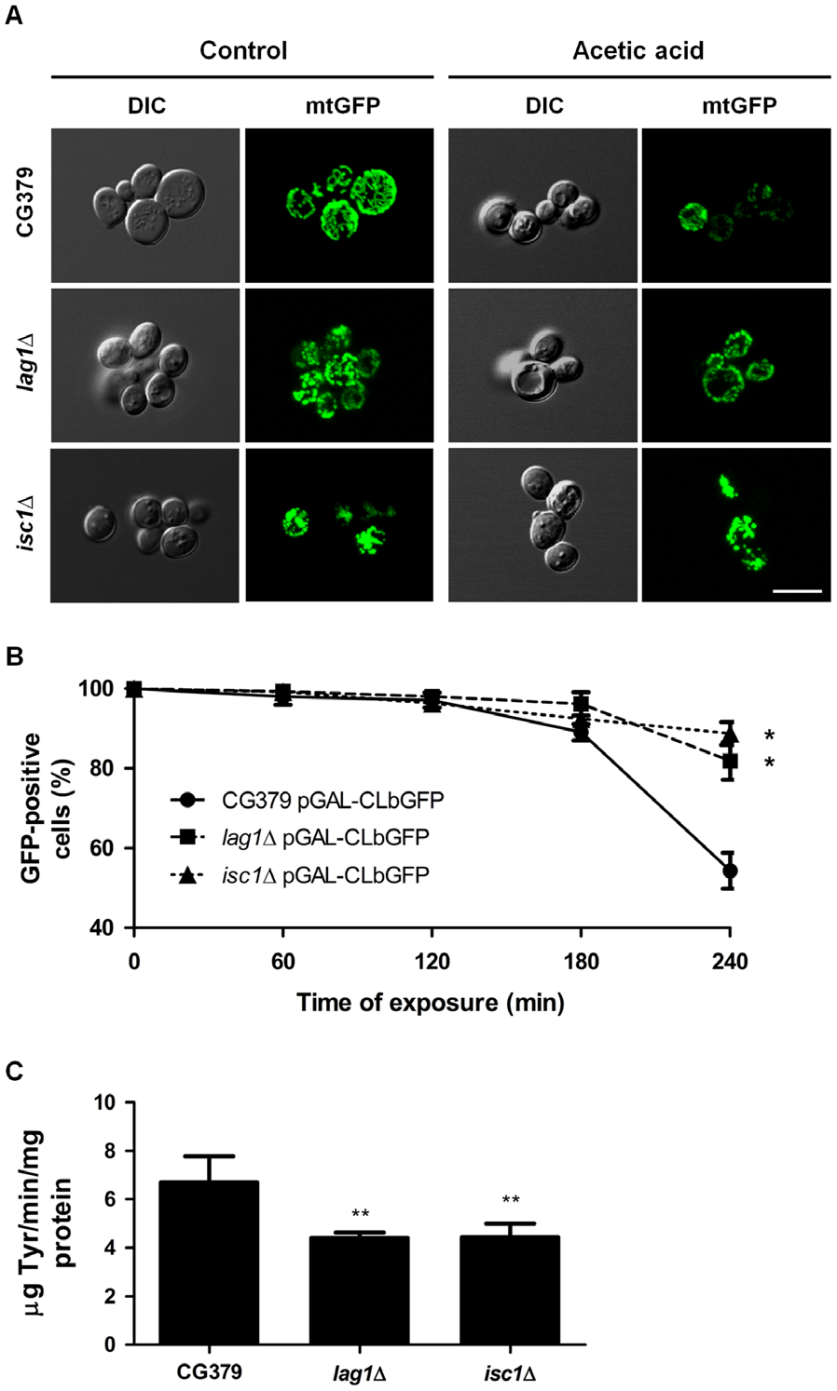
Mitochondrial morphology and degradation. (A) Analysis of mitochondrial morphology in *S. cerevisiae* strains CG379, *lag1*Δ and *isc1*Δ expressing mitochondrial GFP (pGAL-CLbGFP) before (control) and after exposure to 180 mM acetic acid, pH 3.0, for 200 min. Cells were observed by fluorescence microscopy. A representative experiment is shown. Bar, 5 µm. (B) Mitochondrial degradation was assessed in these strains by measuring the loss of mtGFP fluorescence during exposure to 180 mM acetic acid, pH 3.0, up to 240 min. (C) Pep4p activity was detected in extracts of the same strains, by measuring the release of tyrosine-containing acid- soluble peptides from acid-denatured haemoglobin. Values are mean ± SD of at least three independent experiments. Values significantly different from CG379: * P<0.05, ** P<0.01, One-way ANOVA and Tukey Test.

Similar to what has been described in mammalian apoptotic scenarios, the typical yeast mitochondrial morphology changes from a tubular network to a punctuate pattern in response to acetic acid [5], [6]. We thus set out to characterize the relative contribution of sphingolipids to this process. Mitochondrial morphological changes were observed through confocal microscopy using cells transformed with pGal-CLbGFP, a vector that expresses the green fluorescent protein (GFP) fused to a mitochondrial presequence of the citrate synthase that targets GFP to mitochondria (Figure 4A). As expected, under normal conditions, the mitochondrial morphology of the wild-type strain consists of perfect mitochondrial networks. After exposure to acetic acid, mitochondrial networks are destabilized, leading to the formation of the typical punctuate pattern that is normally observed when cells undergo apoptotic cell death. On the other hand, the mitochondrial morphology of untreated *isc1*Δ and *lag1*Δ mutants is distinct from that of the wild-type strain. In these mutant strains, mitochondria formed aggregates under normal conditions, more visible in the *isc1*Δ strain, which were not destabilized by exposure to acetic acid.

Mitochondrial degradation has previously been described in yeast cells exposed to apoptotic stimuli such as acetic acid [5], [6]. In order to address whether deletion of *ISC1* or *LAG1* affected mitochondrial degradation, the loss of mitochondrial GFP (mtGFP) fluorescence, representative of the loss of mitochondrial mass, was monitored by flow cytometry after exposure to acetic acid (Figure 4B). Up to 180 min of treatment, about 90% of cells of all strains still exhibited GFP fluorescence, indicating the levels of mitochondria degradation were low. However, after 240 min of acetic acid treatment, GFP fluorescence was still present in more than 80% of *isc1*Δ and *lag1*Δ mutant cells, whereas only about 50% of wild-type cells exhibited GFP fluorescence. These results suggest that deletion of *ISC1* or *LAG1* delays mitochondrial degradation in response to acetic acid. This phenotype was very similar to that observed with *PEP4*-deleted cells [6]. It is also known that ceramide can activate CatD in mammalian cells [41]. We therefore hypothesized that the delay observed in *isc1*Δ and *lag1*Δ mutant strains could be a result of decreased activity of Pep4p, the yeast CatD orthologue. Indeed, both mutant strains exhibited decreased Pep4p activity (Figure 4C).

### Translocation of cytochrome *c* to the cytosol is impaired in *ISC1* and *LAG1* deleted strains

**Figure 5:**
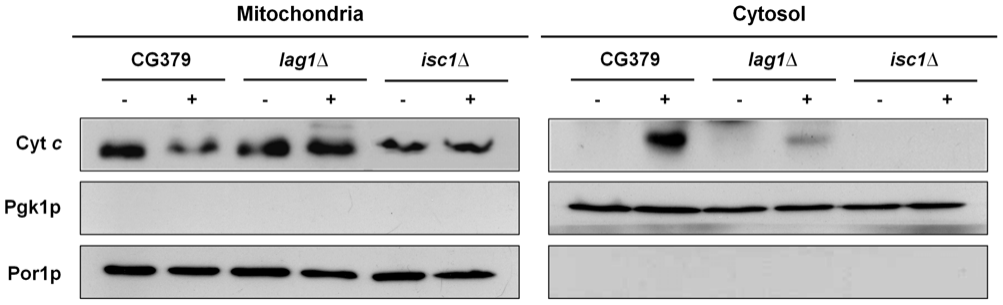
Cytochrome *c* release. Western blot analysis of cytochrome *c* in *S. cerevisiae* strains CG379, *isc1*Δ and *lag1*Δ before (−) and after (+) exposure to 180 mM acetic acid, pH 3.0, for 200 min, in both mitochondrial and cytosolic fractions. Cytosolic phosphoglycerate kinase (Pgk1p) and mitochondrial porin (Por1p) levels were used as loading control of cytosolic and mitochondrial fractions, respectively. A representative experiment is shown of at least two independent experiments with similar results.

A crucial event in yeast apoptosis induced by acetic acid is the translocation of cytochrome *c* from mitochondria to the cytosol. Since we found *isc1*Δ and *lag1*Δ mutant strains were more resistant to acetic acid, we next determined whether acetic acid still triggered release of cytochrome *c* in these strains. Cytosolic and mitochondrial fractions were isolated by differential centrifugation and the integrity of the inner mitochondrial membrane confirmed through determination of the activity of citrate synthase (data not shown), a protein exclusively localized in the mitochondrial matrix. The levels of cytochrome *c* in the different fractions were detected by Western blot (Figure 5). Under normal conditions, cytochrome *c* was detected only in the mitochondrial fractions. As we have previously shown [3], [7], exposure of the wild-type strain to acetic acid resulted in a decrease of the cytochrome *c* content in mitochondria, and consequent detection in the cytosol. In agreement with the phenotype of acetic acid resistance, cytochrome *c* release to the cytosol was significantly decreased or completely suppressed in *lag1*Δ and *isc1*Δ cells, respectively. We also observed that *isc1*Δ cells have a lower overall content of cytochrome *c* than the other strains.

### The resistance of *isc1*Δ and *lag1*Δ cells to acetic acid is associated with lower levels of some ceramide species and exogenous addition of C_2_-phytoceramide sensitizes cells to acetic acid

**Figure 6:**
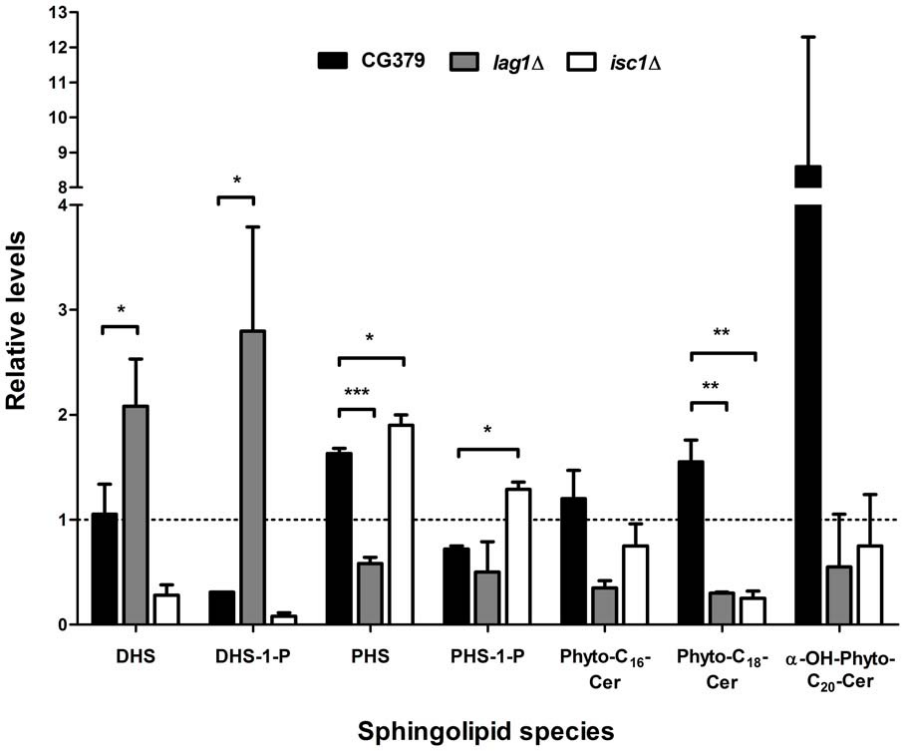
Changes in the levels of sphingolipid species during acetic acid-induced apoptosis. Relative levels of dihydrosphingosine (DHS), dihydrosphingosine-1-phosphate (DHS-1-P), phytosphingosine (PHS), phytosphingosine-1-phosphate (PHS-1-P), phytoceramides (Phyto-C_16_-Cer and Phyto-C_18_-Cer) and α-hydroxylated phytoceramide (α-OH-Phyto-C_20_-Cer) in *S. cerevisiae* CG379 (wild-type), *lag1*Δ and *isc1*Δ strains after exposure to 180 mM acetic acid, pH 3.0, for 200 min are shown. Values are mean ± SD of three independent experiments. * P<0.05, ** P<0.01, One-way ANOVA and Tukey Test. Levels of α-OH-Phyto-C_20_-Cer in CG379 reproducibly displayed a high increase after exposure to acetic acid in all experiments, though relative values varied considerably.

Because *lag1*Δ and *isc1*Δ strains were more resistant to acetic acid than wild-type cells, we speculated that the observed resistance was associated with lower levels of endogenous ceramides. Thus, a lipidomic analysis was performed in *S. cerevisiae* CG379, *lag1*Δ and *isc1*Δ cells untreated or treated with acetic acid for 200 min. The results revealed that several sphingolipid species decreased with acetic acid treatment, some were not affected, and others increased. We observed several distinct patterns between the wild-type strain and the *isc1*Δ and *lag1*Δ mutants (Figure 6). The wild-type strain showed increasing levels of phytosphingosine (PHS) (1.4-fold) upon acetic acid treatment, but dihydrosphingosine-1-phosphate (DHS-1-P) and phytosphingosine-1-phosphate (PHS-1-P) decreased 3.3- and 1.4-fold, respectively. In contrast, DHS-1-P levels did not decrease in *lag1*Δ cells treated with acetic acid and even a 3-fold increase was observed. DHS levels also increased (2-fold) in *lag1*Δ mutants, whereas PHS decreased 1.7-fold. A distinct pattern was observed in *isc1*Δ cells, in which the acetic acid-induced decrease in PHS-1-P levels was suppressed and a small increase (1.3-fold) was observed. Regarding ceramides, the levels of most dihydroceramide and phytoceramide species decreased in wild-type cells treated with acetic acid (data not shown), but increases in some individual phytoceramide species were observed. Notably, these increases were suppressed in both *isc1*Δ and *lag1*Δ mutants. The most notable were changes in α-hydroxy-C_20_-phytoceramide, whose levels increased dramatically in response to acetic acid, and those changes were prevented in both the *isc1*Δ and *lag1*Δ mutants. In line with this observation, cells lacking Sch9p, the yeast orthologue of mammalian protein kinase B, displayed a higher resistance to acetic acid and similar changes in sphingolipid metabolism [42], [43]. Notably, the same authors confirmed that Sch9p plays a critical role in ceramide metabolism by controlling *de novo* ceramide synthesis as well regulating the activity of Isc1p, which is required for ceramide production. These data reinforce the involvement of sphingolipids in the regulation of apoptosis induced by acetic acid.

**Figure 7:**
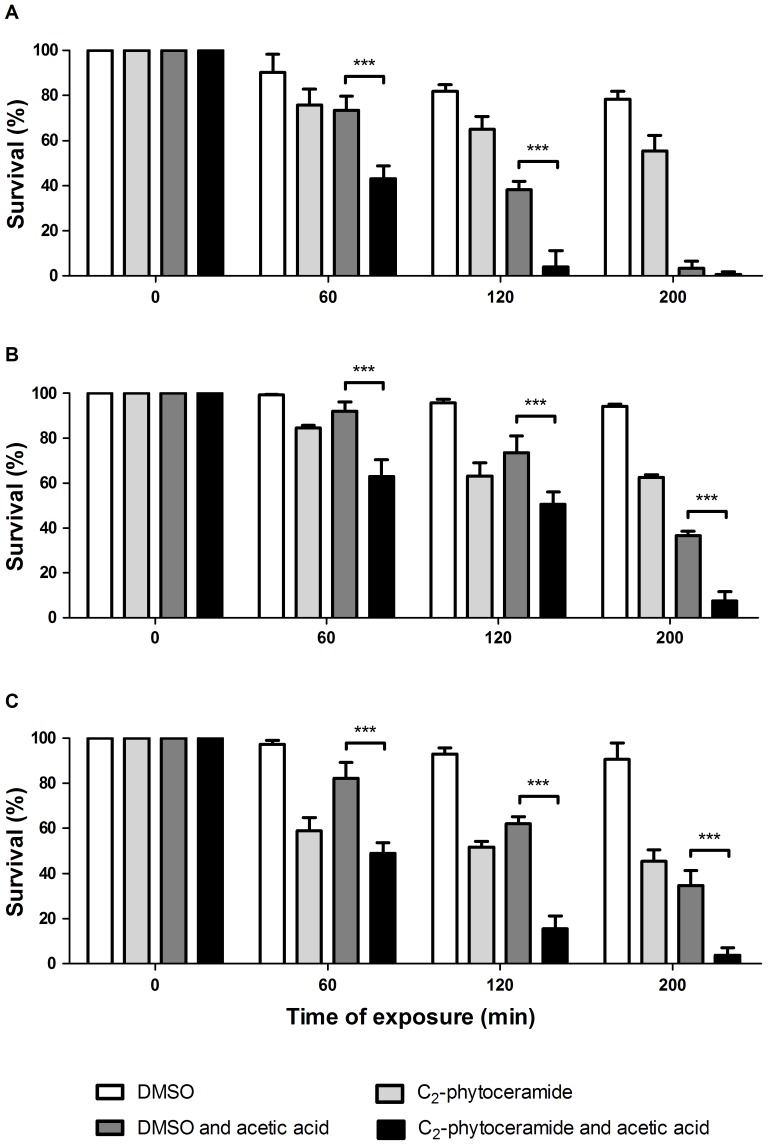
Effect of exogenous C_2_-phytoceramide. Survival of *S. cerevisiae* strain CG379 (A), *lag1*Δ (B) and *isc1*Δ (C) exposed to DMSO, 15 µM of C_2_-phytoceramide, 180 mM acetic acid or 15 µM of C_2_-phytoceramide and 180 mM acetic acid, for up to 200 min. Cell viability was determined by standard dilution plate counts and expressed as a percentage of c.f.u on YPD plates. Values are mean ± SD of at least three independent experiments. Values significantly different: *** P<0.001, One-way ANOVA and Tukey Test.

To further support the hypothesis that the increase in phytoceramide levels is implicated in acetic acid-induced cell death, all strains were exposed to 180 mM of acetic acid and/or 15 µM of C_2_-phytoceramide, a type of ceramide that can have apoptosis-inducing activity [44], or solvent alone (DMSO), for up to 200 min (Figure 7). Combined exposure to C_2_-phytoceramide and acetic acid resulted in significantly greater sensitivity than individual exposure to each agent in both wild-type and *lag1*Δ and *isc1*Δ strains. Interestingly, survival of *lag1*Δ and *isc1*Δ strains simultaneously exposed to acetic acid and C_2_-phytoceramide was similar to that of wild-type cells exposed to acetic acid alone. Thus, not only is phytoceramide sufficient to induce death, but it overcomes the higher resistance seen in the *isc1*Δ and *lag1*Δ mutants.

## Discussion

Several sphingolipids function as lipid second messengers generated in response to different physiological signals and stress stimuli. They affect multiple aspects of cellular function, including apoptosis. In mammalian cells, an increase in ceramide and sphingosine levels promotes apoptosis, whereas an increase in sphingosine-1-phosphate levels inhibits apoptosis [9]. In previous studies, it has been shown that acetic acid triggers a mitochondria-mediated apoptotic pathway associated with mitochondrial dysfunction, such as ROS accumulation, decrease in cytochrome *c* oxidase (COX) activity, and subsequent release of cytochrome *c* [3]. Additionally, other studies implicated the ADP/ATP carrier in the mitochondrial outer membrane permeabilization and cytochrome *c* release [7], and the vacuolar protease Pep4p in mitochondrial degradation [6]. For the first time, this study shows that sphingolipid metabolism plays a role in the mitochondria-mediated yeast apoptotic pathway induced by acetic acid. Deletion of *ISC1*, orthologue of mammalian neutral sphingomyelinase-2, or of *LAG1*, orthologue of mammalian longevity assurance gene (*LASS1*)/ceramide synthase, enhanced the cell survival of yeast cells exposed to acetic acid. Notably, *isc1*Δ and *lag1*Δ mutants exhibited lower levels of some phytoceramide species compared to the wild-type strain as well as higher levels of long-chain base phosphates (PHS-1-P and DHS-1-P, respectively) that have been suggested to exert anti-apoptotic functions. Moreover, exogenous phytoceramide sensitized cells to acetic acid, suggesting it mediates acetic acid-induced apoptosis. This phenotype of the *isc1*Δ mutant contrasts with its higher susceptibility to hydrogen peroxide [26]. However, we have shown that the increased sensitivity of this mutant to hydrogen peroxide is related to the up-regulation of the iron regulon, increased iron uptake, and an enhancement of the Fenton reaction, potentiating oxidative stress. Unlike hydrogen peroxide (that is itself an oxidant), acetic acid perturbs the electron transport chain by specifically affecting the COXII subunit, which binds cytochrome *c*. Therefore, exposure to acetic acid or to hydrogen peroxide should not necessarily lead to the same phenotype. Indeed, we have previously shown that the *aac1/2/3*Δ mutant (a mutant defective in mitochondrial ADT/ATP carrier proteins) is more resistant to acetic acid but more sensitive to hydrogen peroxide, in spite of its defect in cytochrome *c* release in response to both stimuli [7]. Similarly, rho0 mutants are also more resistant to acetic acid and more sensitive to hydrogen peroxide [3], [45].

In mammalian cells, signaling through sphingomyelinases or *de novo* ceramide synthesis is quite distinct. Differences in enzyme subcellular localization, kinetics of activation, and in the levels and species of ceramide generated are some of the features that may explain such a distinct behavior. Sphingomyelinases are essentially localized at the inner and outer leaflet of the plasma membrane and are activated within minutes of stimulation. Moreover, ceramide production by sphingomyelinases is transient and may last only few minutes, while generation by up-regulation of *de novo* synthesis typically takes several hours [46]. Notably, though the yeast orthologues of mammalian sphingomyelinase (Isc1p) and ceramide synthase (Lag1p) have different characteristics from their mammalian counterparts, absence of the yeast sphingomyelinase Isc1p had a higher impact on cell survival than absence of ceramide synthase Lag1p. Therefore, it is tempting to speculate that these distinct signaling pathways also occur in yeast. Though activation of neutral mammalian sphingomyelinases and ceramide synthases has often been implicated in cell death, their role in apoptosis is still poorly understood. Our findings indicate that the yeast model system may allow identifying new direct or indirect targets of the ceramide accumulation triggered during apoptosis.

Several studies have shown that ROS are key signaling molecules in mammalian cells. Accumulation of ROS is directly related with mitochondrial dysfunction and promotion of yeast apoptosis [47]. Accordingly, *isc1*Δ and *lag1*Δ mutant strains accumulated fewer ROS in response to acetic acid than wild-type cells, as well as less mitochondrial dysfunction. Our results also showed that the higher resistance of *isc1*Δ and *lag1*Δ cells is not associated with a decrease in the oxidation levels of any particular protein. In the future, it would be interesting to investigate how Isc1p and Lag1p affect oxidative stress markers during acetic acid treatment, namely lipid peroxidation and levels of antioxidant defenses such as glutathione, superoxide dismutase and catalase activities. Although a previous study showed that exposure to acetic acid did not alter catalase or superoxide dismutase activity in wild-type cells, acetic acid cell death decreased in cells overexpressing catalase T and increased when Cu,Zn-superoxide dismutase was overexpressed, suggesting that decreasing the levels of hydrogen peroxide protects cells from acetic acid-induced cell death, and vice versa [48].

In mammalian cells, ceramides play a role in ROS production and MOMP. It has been shown that ceramides increase ROS production by directly inhibiting the mitochondrial complex III [13], and increasing the permeability of mitochondrial membranes to cytochrome *c* through the formation of pores [14]. Notably, previous studies showed that acetic acid elicits similar mitochondrial dysfunctions in yeast, specifically affecting mitochondrial COX activity [3] and triggering MOMP and cytochrome *c* release [7]. Our results show that the increase in ROS levels and cytochrome *c* release into the cytosol triggered by acetic acid was significantly reduced in yeast cells deficient in the Lag1p ceramide synthase subunit or in Isc1p. In mammalian cells, the Bcl-2 protein family plays a central role in MOMP and subsequent cytochrome *c* release. The mechanism by which MOMP occurs is still under study but, among other possibilities, has been attributed to the formation of pores in the outer mitochondrial membrane by Bax and Bak. More recently, formation of ceramide channels has been proposed as another mechanism mediating the release of pro-apoptotic proteins from mitochondria during the induction phase of apoptosis [49], [50]. Studies using yeast mitochondria and planar phospholipid membranes have shown that activated Bax interacts synergistically with ceramide channels promoting MOMP [51]. Conversely, anti-apoptotic Bcl-2 family proteins disassemble ceramide channels in yeast [31]. Subsequently, it was described that sphingosine-1-phosphate and hexadecenal act as specific cofactors of BAX/BAK activation in mammalian cells, enhancing MOMP and cytochrome *c* release [15]. Therefore, it is conceivable that several sphingolipid species control mitochondrial apoptosis in yeast as well as in mammals. Our results suggest that, as proposed in mammalian cells, ceramide has an active role in cytochrome *c* release in yeast in response to apoptotic insult, possibly through the formation of ceramide pores. Whether the decrease in cytochrome *c* release observed in the absence of Isc1p and Lag1p is related with changes in the mitochondrial level of ceramide and with its propensity to form ceramide pores warrants further investigation.

How would Lag1p and Isc1p, located in the ER, cause ceramides to accumulate in mitochondria of wild-type cells during acetic acid stress? Isc1p localizes to mitochondria in the post-diauxic phase of cultures grown in glucose medium, when cell metabolism shifts from fermentation to respiration [20]. Under the respiratory conditions used in this study (cells grown in galactose medium to exponential phase), Isc1p probably localizes to mitochondria. Moreover, it has been shown that ceramide exchanges between mitochondria-associated membranes (ER-like membranes) and mitochondria overcomes the need for mitochondria ceramide synthesis to increase ceramide to levels sufficient to cause MOMP [52]. Therefore, mitochondrial permeabilization is not completely dependent on ceramide production in mitochondria.

It has been suggested that mitochondrial fragmentation is required for MOMP and cytochrome *c* release. In cardiomyocytes, ceramide increases the mitochondrial content of Drp-1 and Fis1 proteins, which are involved in mitochondrial fission, promoting the fragmentation of the mitochondrial network and apoptosis [53]. The yeast orthologue of human Drp-1 has also been implicated in mitochondrial fragmentation/degradation and cell death following several death stimuli, including acetic acid [5]. We observed that acetic acid leads to mitochondrial fragmentation in wild-type cells as described. However, in *isc1*Δ and *lag1*Δ cells, mitochondria formed aggregates that were not destabilized by acetic acid treatment. Our results therefore suggest that ceramides generated by Isc1p and Lag1p are implicated in normal mitochondrial function and in morphological changes that may enhance cytochrome *c* release into the cytosol. We have previously observed that exposure to acetic acid leads to mitochondria clusters in *aac1/2/3*Δ and *pep4*Δ mutant cells, but not in *atp2*Δ mutant cells, indicating this phenotype is not due low mitochondrial energetic levels. However, in the latter case, the mitochondrial morphology of the mutants before treatment was indistinguishable from that of wild-type cells [6]. Sphingolipid metabolism had previously been associated with mitochondrial morphology since overexpression of *YDC1* results in decreased ceramide levels and increased mitochondrial fragmentation [32]. However, it remains to be established how changes in sphingolipid homeostasis affect mitochondrial morphology, both before and after exposure to acetic acid. It is therefore possible that ceramide or a ceramide-regulated protein increases mitochondrial fission by activating or recruiting Drp1p and Fis1p to this organelle.

In mammalian cells, several proteins have been identified as ceramide targets, including the endossomal acidic aspartic protease CatD. Ceramide generated by the acid sphingomyelinase binds to CatD, inducing the autocatalytic proteolysis of the pro-enzyme to its active form [41]. The yeast Pep4p, with homology to CatD, translocates from the vacuole to the cytosol in response to several stress conditions. It is essential for the removal of oxidized proteins after oxidative damage induced by H_2_O_2_ or chronological ageing [54], and is involved in mitochondrial degradation in acetic acid-induced apoptosis [6]. Deletion of *PEP4* results in increased sensitivity to acetic acid that apparently is not due to an accumulation of oxidized proteins [6]. Our results suggest that the decrease in acetic acid-induced mitochondrial degradation in *isc1*Δ and *lag1*Δ cells is associated with a lower release/activation of Pep4p. Curiously, the resistance phenotype of *aac1/2/3*Δ to acetic acid is also associated with a decrease in mitochondrial degradation and cytochrome *c* release. However, defects in mitochondrial degradation do not always correlate with increased resistance to acetic acid. Indeed, cells lacking Pep4p have increased sensitivity to acetic acid that is associated with a delay in mitochondrial degradation, indicating removing damaged mitochondria may have a protective role in acetic acid-treated cells. The higher resistance of *aac1/2/3*Δ, *isc1*Δ and *lag1*Δ mutants to acetic acid, in comparison with the sensitivity of *pep4*Δ cells, is probably due to defects in the release of cytochrome *c*. Moreover, *ISC1*-deleted strains are defective in aerobic respiration due to an inability to up-regulate genes required for growth in non-fermentable carbon sources [22]. It has been shown that respiratory-deficient mutants are more resistant to acetic acid-induced apoptosis [3], which is consistent with the increased resistance of *isc1*Δ cells to acetic acid-induced apoptosis.

It has been shown that the MAPK of the HOG pathway, Hog1p, is activated in response to acetic acid and phosphorylates the aquaglyceroporin Fps1p, ultimately leading to its ubiquitination and removal from the plasma membrane. Intracellular accumulation of acetate is reduced when Fps1p is depleted, leading to increased cellular resistance to acetic acid [55]. Since *ISC1* deletion results in Hog1p activation [56], we questioned if the higher resistance of *isc1*Δ cells to acetic acid-induced apoptosis could be due to decreased acetic acid transport and accumulation. We therefore measured acetic acid initial uptake rates and intracellular accumulation using ^14^C-labelled acid. However, *isc1*Δ cells did not show a decrease in transport rates or in intracellular levels of acetate when compared with their wild-type counterpart (data not shown), indicating that is not the case.

In summary, while differences in ceramide metabolism exist between yeast and mammalian cells, our study indicates that ceramide production contributes to a mitochondria-mediated apoptotic cell death, especially through hydrolysis of complex sphingolipids and *de novo* synthesis catalyzed by Isc1p and Lag1p, and offers new perspectives for enhanced understanding of how ceramide metabolism impacts MOMP and apoptosis. The importance of ceramide in mammalian cell death is increasingly apparent, and thus our study further supports the use of yeast as a valuable model to investigate the regulation of programmed cell death by ceramide.

## Materials and Methods

### Strains and Plasmids

All *Saccharomyces cerevisiae* strains used in this study are listed in Table 1. *S. cerevisiae* strain CG379 (closely related to S288c background; Craig N. Giroux personal communication) was used as the wild-type strain. The *lac1*Δ, *lag1*Δ, *ydc1*Δ, *ypc1*Δ and *isc1*Δ mutants were constructed in CG379 by homologous recombination using disruption cassettes (KanMX4) amplified by Polymerase Chain Reaction (PCR), with the oligonucleotides listed in Table 2 (numbers 1–10) and genomic DNA isolated from the respective mutants of the yeast strain BY4741 (Euroscarf collection, Germany).

**Table 1:** List of *S. cerevisiae* strains used in this study.

Strain	Genotype	Reference/Source
**CG379**	Matα, *ade5*, *his2*, *leu2-112*, *trp1-289*, *ura3-52*	Yeast Genetic Stock Center, University of California, USA
**CG379 pYES2**	CG379 harboring pYES2	This study
**CG379 pGAL-CLbGFP**	CG379 harboring pGAL-CLbGFP	This study
***lac1*** **Δ**	CG379 *lac1*Δ :: *KanMX4*	This study
***lag1*** **Δ**	CG379 *lag1*Δ :: *KanMX4*	This study
***lag1*** **Δ pYES2**	*lag1*Δ harboring pYES2	This study
***lag1*** **Δ pYES2-** ***LAG1***	*lag1*Δ harboring pYES2-*LAG1*	This study
***lag1*** **Δ pGAL-CLbGFP**	*lag1*Δ harboring pGAL-CLbGFP	This study
***ydc1*** **Δ**	CG379 *ydc1*Δ :: *KanMX4*	This study
***ypc1*** **Δ**	CG379 *ypc1*Δ :: *KanMX4*	This study
***ypc1*** **Δ pYES2**	*ypc1*Δ harboring pYES2	This study
***isc1*** **Δ**	CG379 *isc1*Δ :: *KanMX4*	This study
***isc1*** **Δ pYES2**	*isc1*Δ harboring pYES2	This study
***isc1*** **Δ pYES2-** ***ISC1***	*isc1*Δ harboring pYES2-*ISC1*	This study
***isc1*** **Δ pGAL-CLbGFP**	*isc1*Δ harboring pGAL-CLbGFP	This study

**Table 2:** List of oligonucleotides used in this study.

Number	Name	Oligonucleotide Sequence (5′→3′)
1	**Lac1Fw**	GGAGGGAGAAAGTATTGGAATCT
2	**Lac1Rv**	GAAAGCACTAACATCAACATGGA
3	**Lag1Fw**	CGTCATCTTCCATTTGAAATCC
4	**Lag1Rv**	TCTTACTAGGAGTCTTGGCGAGA
5	**Ydc1Fw**	TGTCCGATAGCGTACGCCA
6	**Ydc1Rv**	GCCGGTTTTCCAAGCAG
7	**Ypc1Fw**	CGCGAGACATCGGAAAATA
8	**Ypc1Rv**	CATGTCCCGAATTAGCTAACAA
9	**Isc1Fw**	AGGTCGACTGCCGTCTAGAT
10	**Isc1Rv**	GCGGACTTCATTTTACTCCAGAC
11	**Lac1KanFw**	TGGGCATTGTACCTGATCATG
12	**Lac1KanRv**	GGCCTACTATGACAACGATAGCT
13	**Lag1KanFw**	CCAGTCCGTCAAGACTAATATCG
14	**Lag1KanRv**	CGATGATTCATTGAGATCTGTCA
15	**Ydc1KanFw**	AAATCCCTCGTTCCCGG
16	**Ydc1KanRv**	TATGTGCCGCCGACATG
17	**Ypc1KanFw**	GGACGGATTATCACGCAAGT
18	**Ypc1KanRv**	CAGAAGCCAAAATAGCATTCAA
19	**Isc1KanFw**	TTGCAGCAGCGAGTCCA
20	**Isc1KanRv**	CGAACGAGGCAGTAGTCATGTT
21	**KanRv**	AATCGAATGCAACCGGC
22	**LAG1_HindIII_Fw**	ACGAC**AAGCTT**AACATGACATCAGCTACGGACAAAT
23	**LAG1_XhoI_Rv**	AGATA**CTCGAG**CGTTTATTCACACTTTTCCTTAGAT

Restriction sites are marked in bold in respective oligonucleotide sequence.

CG379 cells were transformed by electroporation and transformants selected on rich medium [YPD; 1% (w/v) Yeast extract, 2% (w/v) Bactopeptone and 2% (w/v) Glucose] containing 200 µg/mL geneticin. The correct integration of the disruption cassettes was confirmed by PCR using oligonucleotides (numbers 11–20) that bind upstream and downstream of the insertion, plus an additional oligonucleotide (number 21) binding within the kanamycin gene. In addition, *lag1*Δ and *isc1*Δ were transformed by electroporation with pYES2-*LAG1* and pYES2-*ISC1* vectors, respectively. For mitochondrial studies, wild-type, *lag1*Δ and *isc1*Δ strains were transformed with pGAL-CLbGFP.

All the plasmids used in this study are listed in Table 3. The *LAG1* gene was amplified by PCR from genomic DNA isolated from the CG379 strain using the oligonucleotides LAG1_HindIII_Fw (number 22) and LAG1_XhoI_Rv (number 23), which introduce *Hin*dIII and *Xho*I restriction sites in the flanks, and cloned into pYES2 using these enzymes, resulting in pYES2-*LAG1*.

**Table 3:** List of plasmids used in this study.

Plasmid	Description	Reference/Source
**pYES2**	*URA3*; *P_GAL1_*	Invitrogen
**pYES2-** ***LAG1***	*LAG1* inserted in pYES2	This study
**pYES2-** ***ISC1***	*ISC1* inserted in pYES2	[19]
**pGAL-CLbGFP**	*URA3, P_GAL1_*_/*10*_, Presequence of the mitochondrial citrate synthase (*CIT1*) fused to *GFP*	[62]

### Growth conditions and cell death assays

Strains were grown in Synthetic Complete Galactose medium [SC Gal; 2% (w/v) Galactose, 0.67% (w/v) Yeast nitrogen base without aminoacids, 0.14% (w/v) Drop-out mixture lacking histidine, leucine, tryptophan and uracil, 0.008% (w/v) Histidine, 0.04% (w/v) Leucine, 0.008% (w/v) Tryptophan and 0.008% (w/v) Uracil] to early exponential phase (OD_600_ = 0.5–0.6) at 26°C in an orbital shaker at 140 rpm, with a ratio of flask volume/medium of 5∶1. Strains transformed with plasmids were grown in the same medium lacking uracil. Solid media were prepared by adding 2% (w/v) agar. Galactose was used as the carbon and energy source to address mitochondrial function, as this leads to higher mitochondrial mass because galactose is less effective in the repression of respiratory metabolism [57]. For acetic acid and C_2_-phytoceramide treatments, strains were cultured under the conditions described above, harvested and suspended in SC Gal at pH 3.0 (set with HCl) containing 180 mM of acetic acid (Panreac, Spain) and/or 15 µM of N-acetyl-D-phytosphingosine (C_2_-phytoceramide; Sigma Aldrich) or solvent alone (DMSO) for up to 200 min. Cell viability was measured as a percentage of colony forming units (c.f.u.) on YPD medium.

### ROS quantification

Intracellular superoxide anion and mitochondrial ROS were detected by flow cytometry using Dihydroethidium (DHE) and MitoTracker Red CM-H_2_XRos (Molecular Probes, Eugene, U.S.A.) as probes, respectively. For DHE staining, untreated or acetic acid-treated cells (180 mM) of wild-type and mutant cells were harvested by centrifugation, resuspended in 500 µL PBS [80 mM Na_2_HPO4, 20 mM NaH_2_PO4 and 100 mM NaCl)] and incubated with 5 µg/mL DHE for 30 min in the dark. For MitoTracker Red CM-H_2_XRos staining, untreated or acetic acid treated cells (180 mM) of wild-type and mutant cells were harvested, resuspended in PBS and incubated with 0.4 µg/mL MitoTracker Red CM-H_2_XRos at 37°C for 20 min in the dark. Cells with red fluorescence [FL-3 channel (488/620 nm)] were considered to contain superoxide anion or mitochondrial ROS.

### Mitochondrial morphology

Mitochondrial morphology changes were observed using cells transformed with a plasmid expressing mitochondrial GFP (pGAL-CLbGFP). After exposure to acetic acid, images of the mitochondrial network were acquired in an Olympus BX61 microscope equipped with a confocal Olympus FLUOVIEW microscope with an Olympus PLAPON 60×/oil objective with a numerical aperture of 1.42, and using the Olympus FLUOVIEW software. Mitochondrial degradation was also determined in these cells by assessing the percentage of cells that still exhibit mtGFP fluorescence after exposure to acetic acid, using a flow cytometer. The percentage of cells that exhibit GFP fluorescence was determined in biparametric histograms [ratio (FL-1 area (log)/FS (log))×GFP fluorescence (FL-1 Peak)] to eliminate variations in fluorescence due to cell size and to discriminate between the cells with intense spots of mitochondrial-GFP and cytosolic-GFP resultant from mitochondrial degradation.

### Pep4p activity assay

Cells were grown as described above, and harvested at the exponential phase (OD_600_ = 0.7–0.9). Yeast extracts were prepared in 0.1 M Tris, pH 7.6, by vigorous shaking of the cell suspension in the presence of glass beads, for 5 min. Short pulses of 1 min were used, with 1 min intervals on ice. Pep4p activity was determined using 0.250 mg total protein, by measuring the release of tyrosine-containing acid-soluble peptides from acid-denatured haemoglobin [expressed as µg Tyr min^−1^ (mg protein)^−1^] [58].

### Cytochrome *c* detection

For cytochrome *c* detection, one litter of cells were grown and treated under the conditions described above, harvested at the end of exponential phase (OD_600_ = 1.4–1.6) and mitochondrial and cytosolic fractions were prepared as described [7]. Estimation of the protein concentration of the fractions was determined by the Bradford method using BSA as standard [59]. The integrity of mitochondria during the procedure was evaluated by measuring citrate synthase activity [7]. Mitochondrial and cytosolic fractions were separated electrophoretically on a 12.5% SDS-polyacrylamide gel and transferred to a Hybond-P Polyvinylidene difluoride membrane (PVDF; GE Healthcare) at 0.8 mA/cm^2^ during 1 h. Membranes were cut into strips and incubated with the primary antibodies mouse monoclonal anti-yeast phosphoglycerate kinase (PGK1) antibody (1∶5000, Molecular Probes), mouse monoclonal anti-yeast porin (POR1) antibody (1∶5000, Molecular Probes) and rabbit polyclonal anti-yeast cytochrome *c* (CYC1) antibody (1∶1000, custom-made by Millegen), followed by incubation with secondary antibodies against mouse or rabbit IgG-peroxidase (1∶5000; Sigma Aldrich). Pgk1p and Por1p were used as a loading control for cytosolic and mitochondrial fractions, respectively. Immunodetection of bands was revealed by chemiluminescence (ECL, GE Healthcare).

### Sphingolipid profiling

Levels of phytoceramide, dihydroceramide, α-hydroxylated phytoceramides, PHS, PHS-1-P, DHS and DHS-1-P were measured by the high-performance liquid chromatography/mass spectrometry (LC-MS/MS) methodology as previously described [60]. Analytical results of lipids were expressed as lipid level/total cell number. The strains were grown in 250 mL of medium and treated with acetic acid under the conditions described above and harvested at the early of exponential phase (OD_600_ = 0.5–0.6). The pellets were re-suspended in 1 mL of a lipid extraction solvent containing: Iso-propanol (50%), Ethanol (10%), Pyridine (2%), Ammonia (25%), Water (15%), acid washed glass beads 425–600 µm were added. The cells were then treated as described in [61]. Modification to the method, was vortexing step was performed as follow: 3 minutes on, 1 minute off, at 4°C for 5 times repeats. Lipids were normalized to cell number.

### Flow cytometric assays

All the flow cytometric assays were performed in an Epics® XL™ (Beckman Coulter) flow cytometer, equipped with an argon-ion laser emitting a 488-nm beam at 15 mW. The population of cells with high homogeneity and frequency was gated in a histogram of Side Scatter (SS)×Forward Scatter (FS). Twenty thousand cells per sample were analyzed. The resulting data were analyzed with WinMDI 2.8 software.

### Reproducibility and statistic analysis of the results

The results obtained are represented by mean and standard deviation (SD) values of at least three independent experiments. Statistical analyses were carried out using GraphPad Prism Software v5.00 (GraphPad Software, California, USA). P-values lower than 0.05 were assumed to represent a significant difference.

## Supporting Information

Figure S1 **Protein oxidation in** ***S. cerevisiae*** **CG379 (wild-type),** ***lag1*** **Δ and** ***isc1*** **Δ strains before (−) and after (+) exposure to 180 mM acetic acid, pH 3.0, for 200 min.** Yeast extracts were prepared in 50 mM potassium phosphate buffer (pH 7.0) containing protease inhibitors (Complete, Mini, EDTA-free Protease Cocktail Inhibitor Tablets; Boehringer Mannhein), by vigorous shaking of the cell suspension in the presence of glass beads for 5 min. Cell debris was removed by centrifugation at 13000 rpm for 15 min and protein content was determined by the method of Lowry, using bovine serum albumin as a standard. Proteins (15 µg) were derivatized with dinitrophenylhydrazine, as described [54], and fractionated by SDS-PAGE using 12.5% gels. (A) Western blotting analysis of protein carbonylation. After electrophoresis, proteins were electroblotted onto a nitrocellulose membrane (Hybond-ECL, GE Healthcare) and immunodetected using rabbit IgG anti-DNP (Sigma, St. Louis, MO, USA) at a 1∶5000 dilution, as the primary antibody, and goat anti-rabbit IgG-peroxidase (Sigma, St. Louis, MO, USA) at a 1∶5000 dilution, as the secondary antibody. Immunodetection was performed by chemiluminescence, using a kit from GE Healthcare (RPN 2109). (B) Silver staining of a replica gel. C+, CG379 cells treated with 1.5 mM H_2_O_2_ for 200 min. A representative experiment of at least two independent experiments with similar results is shown.
(TIF)
